# Reframing obesity through the gut microbiota: functional dysbiosis and metabolic disease

**DOI:** 10.1097/MCO.0000000000001230

**Published:** 2026-05-18

**Authors:** Alaa Hamdan, Ziad Al Nabhani

**Affiliations:** a Department of Visceral Surgery and Medicine, Bern University Hospital; b Maurice Müller Laboratories, Department for Biomedical Research, University of Bern, Bern, Switzerland

**Keywords:** diet-induced dysbiosis, fecal microbiota transplantation, gut microbiota, obesity, prebiotics

## Abstract

**Purpose of review:**

Obesity and its metabolic complications remain major global health challenges. Beyond excess caloric intake, emerging evidence implicates diet-induced gut microbiota dysfunction as a modulator of metabolic homeostasis. This review examines recent advances in understanding how functional alterations of the gut microbiota contribute to obesity pathogenesis.

**Recent findings:**

Current data indicate that obesity is characterized less by specific microbial taxa and more by disruption of key microbial functions. Diet-induced dysbiosis alters short-chain fatty acid production, bile acid metabolism, tryptophan-derived signaling, and intestinal barrier integrity. These changes promote metabolic endotoxemia, impair enteroendocrine hormone secretion, and disrupt gut–brain and gut–liver communication, contributing to adipose tissue inflammation, hepatic steatosis, and insulin resistance. Experimental and clinical studies further suggest that microbiota-targeted interventions, including dietary fiber enrichment, prebiotics, synbiotics, and fecal microbiota transplantation, can partially restore microbial metabolic function and improve selected metabolic outcomes.

**Summary:**

Obesity is increasingly conceptualized as a state of diet-driven functional gut microbiota disruption. Targeting microbial metabolic pathways rather than individual taxa may offer a promising adjunctive strategy to complement established therapies for obesity-related metabolic disease.

## INTRODUCTION

The global prevalence of obesity and related metabolic disorders, including type 2 diabetes, continues to rise at an alarming rate. Obesity is fundamentally characterized by a sustained positive energy balance; however, the biological systems regulating energy intake, expenditure, and nutrient absorption are complex and tightly controlled by integrated neural, endocrine, metabolic, and immune networks. Despite widespread recommendation of lifestyle interventions, long-term weight control remains difficult to achieve, highlighting gaps in our understanding of the mechanisms that sustain metabolic dysfunction.

In recent years, the gut microbiota has emerged as a key environmental modulator of host metabolism. This diverse microbial ecosystem actively participates in nutrient digestion, bile acid transformation, immune regulation, and enteroendocrine signaling [1]. Disturbances in microbial composition and function, collectively termed dysbiosis, have been consistently associated with obesity and insulin resistance [2,3]. However, accumulating evidence suggests that obesity is not defined by the presence or absence of specific microbial taxa. Rather, it reflects a disruption of microbial functions that normally preserve metabolic flexibility, regulate energy harvest, maintain gut barrier integrity, and coordinate endocrine signaling [4,5].

Diet represents the principal driver of microbiota composition and activity. Western dietary patterns rich in saturated fatty acids and refined carbohydrates rapidly and reproducibly remodel microbial communities, shifting metabolite production, bile acid pools, and host–microbe interactions [1,6–8]. Under physiological conditions, commensal microbes support energy homeostasis and immune tolerance. In contrast, chronic exposure to obesogenic diets promotes a functional imbalance characterized by impaired short-chain fatty acid (SCFA) signaling, altered bile acid metabolism, increased intestinal permeability, and low-grade systemic inflammation. These alterations collectively converge on adipose tissue, liver, and central appetite regulation pathways, reinforcing the obese phenotype. Thus, obesity can be conceptualized not solely as a disorder of excess caloric intake, but as a state of diet-driven microbial functional disruption that amplifies metabolic inflammation and hormonal dysregulation.

Animal models have provided critical mechanistic insights into microbiota–host interactions and have been instrumental in establishing causal relationships that cannot be readily assessed in humans [9,10]. Rodent models, however, differ from humans in metabolic physiology, immune responses, and microbiota composition, and often rely on controlled dietary interventions such as high-fat diets that do not fully capture the complexity of human dietary patterns. When integrated with emerging clinical and human multiomics data, these preclinical findings offer a powerful framework to better understand microbiota–host interactions in obesity.

This review synthesizes recent advances in understanding the mechanistic links between diet-induced gut microbiota dysbiosis and obesity. We focus on integrated pathways involving energy harvest, metabolic endotoxemia, enteroendocrine signaling, and adipose–liver crosstalk, and discuss emerging microbiota-targeted strategies aimed at restoring metabolic homeostasis. 

**Box 1:**
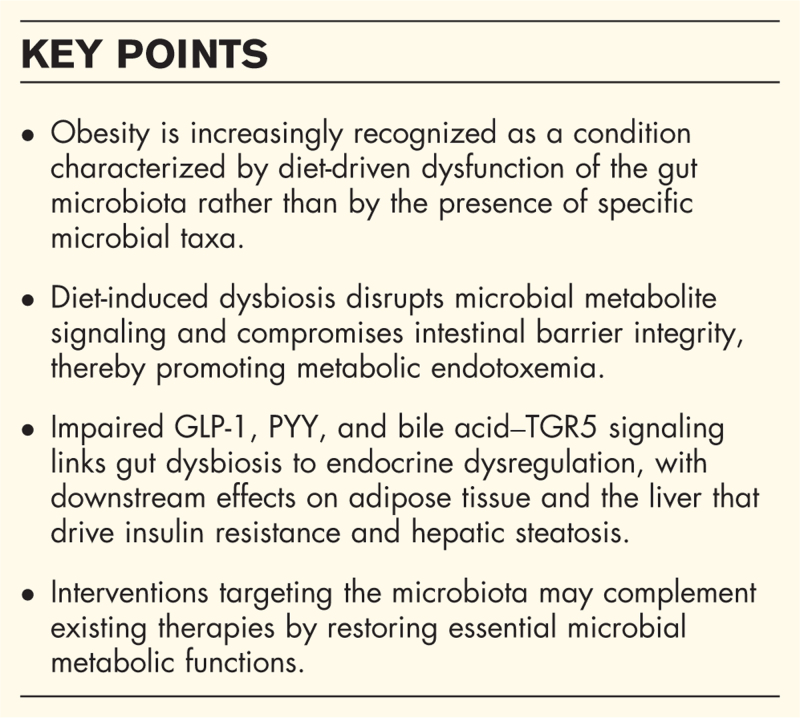
no caption available

## DIET-INDUCED GUT MICROBIAL DYSBIOSIS IN OBESITY

The gut microbiota is shaped by various host and environmental factors, including age, genetics, mode of delivery, lifestyle, and overall health. Among these, diet is the principal factor influencing microbial composition. Modern dietary patterns, such as the Western diet high in sugar and rich in saturated fats and animal products, are potent drivers of microbial shifts [6]. The responsiveness of the gut microbiota to dietary intake has been demonstrated in controlled human dietary interventions, showing that short-term consumption of an animal-based diet rich in saturated fat or plant-based, high-fiber diets can rapidly and reproducibly alter the gut microbiome [7]. Microbial community structure undergoes significant shifts within 24–48 h following dietary changes, with animal-based diets causing more pronounced alterations. These changes include the enrichment of bile-tolerant taxa such as *Bilophila*, *Bacteroides*, and *Alistipes*, and the depletion of polysaccharide-fermenting Firmicutes, including *Roseburia* and *Eubacterium rectale*[7]. Furthermore, these compositional shifts correspond with functional changes in microbial metabolism, characterized by increased amino acid fermentation and decreased production of carbohydrate-derived SCFAs [7]. Consistent with the rapid and reversible diet-driven remodeling of the human gut microbiome observed in short-term interventions, murine models and human studies associate obesity with sustained alterations in gut microbiota composition and function compared to healthy controls. Prolonged high-fat feeding in mice induces expansion of mucus-degrading taxa, depletion of barrier-protective genera such as *Akkermansia* and compromised intestinal barrier integrity thereby promoting pro-inflammatory and metabolically adverse host–microbe interactions [11]. Notably, however, diet-induced obesity develops heterogeneously across hosts. Obesity-resistant (OR) mice display a distinct protective microbiota signature marked by elevated abundance of RF39 lineage and *Lactobacillus*, alongside reduced *Helicobacter* and *Rothia*. This signature correlates with reduced intestinal fatty acid uptake and attenuates hepatic triglyceride accumulation and resistance to steatosis despite prolonged high fat diet (HFD) exposure [12]. Human studies support a link between obesity-associated metabolic deterioration and microbiome disruption. Individuals with obesity and type 2 diabetes (T2D) exhibit more pronounced dysbiosis than those with obesity and preserved glucose tolerance [13]. Complementing these findings, a large-scale cohort multiomics study identified over 500 circulating metabolites associated with impaired glucose regulation. Approximately one-third of these metabolites associated with gut microbiota features and were modifiable through lifestyle interventions, including dietary changes and physical activity [14^▪▪^]. These findings support the presence of a functional microbiome-metabolome axis that significantly influences insulin resistance. Collectively, recent evidence indicates that obesity is characterized not by a single disease-specific taxonomic signature but by convergent disruptions in microbial functions driven by dietary factors closely linked to metabolic dysfunction.

## GUT MICROBIOTA-MEDIATED MECHANISMS IN OBESITY

**FIGURE 1:**
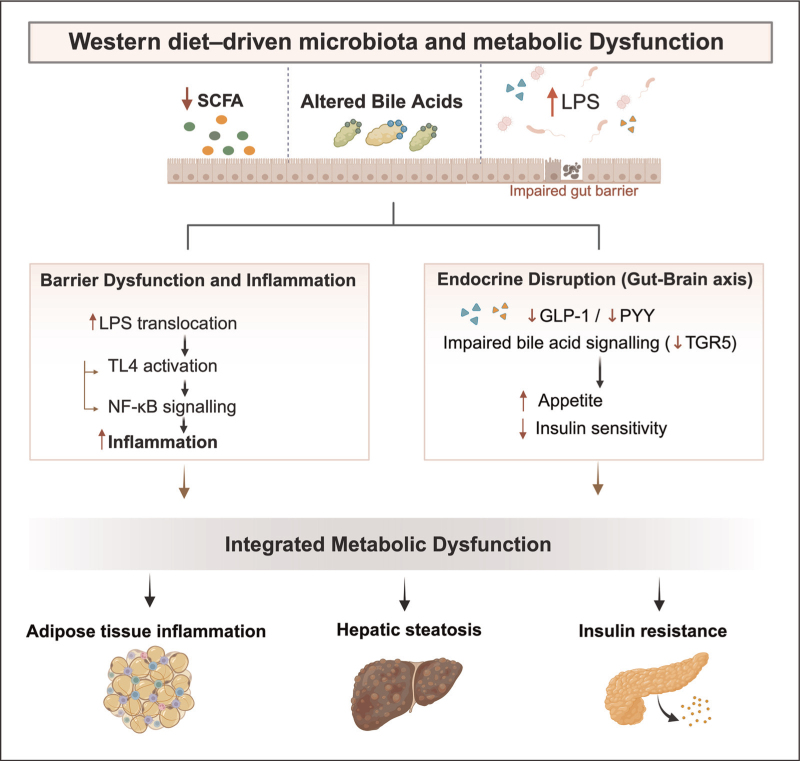
Western diet-driven microbiota and metabolic dysfunction. Western dietary patterns induce functional alterations in the gut microbiota, characterized by reduced short-chain fatty acid (SCFA) production, altered bile acid composition, and increased lipopolysaccharide (LPS) exposure. These changes impair intestinal barrier integrity, increasing gut permeability and promoting metabolic endotoxemia through activation of the Toll-like receptor 4 (TLR4)–NF-κB signaling pathway. Concurrently, endocrine dysfunction occurs, marked by decreased glucagon-like peptide-1 (GLP-1) and peptide YY (PYY) secretion, as well as impaired bile acid–Takeda G-protein-coupled receptor 5 (TGR5) signaling. The crosstalk between inflammatory and enteroendocrine pathways contributes to adipose tissue inflammation, hepatic steatosis, and systemic insulin resistance. This figure was created using BioRender.

Beyond compositional shifts induced by dietary patterns, the gut microbiota serves as a functional orchestrator of host metabolism, contributing to obesity through interconnected mechanisms involving enhanced energy harvest, metabolic endotoxemia, and dysregulated endocrine signaling [9,15] (Fig. 1). At the forefront of this interaction is the energy harvest hypothesis, which proposes that an obesity-associated gut microbiota harbors an expanded enzymatic repertoire for fermenting otherwise indigestible dietary fibers, thereby promoting greater energy extraction and storage while contributing to appetite dysregulation and a positive energy balance independent of differences in caloric intake. This concept, originating from foundational preclinical demonstrations of transmissible increased energy harvest capacity [16], continues to gain mechanistic support. HFD-induced shifts in mice enhance Firmicutes dominance and metabolic efficiency, thereby favoring caloric extraction [11]. In humans, intestinal energy harvest partly mediates microbiota-associated weight outcomes following bariatric surgery, providing compelling evidence that microbiota composition influences effective caloric absorption in clinical contexts [17], and fecal microbiota transplantation from lean donors sustains microbiome alterations linked to reduced adiposity and improved energy balance in obese adolescents [18^▪▪^]. Although direct energy harvest contributes modestly to the caloric surplus in obesity, typically accounting for only 2–5% of energy demand in Western diets, as quantified by recent fermentation analysis [19].

Nevertheless, this hypothesis remains foundational for understanding microbial influences on energy balance. Accordingly, emerging evidence increasingly emphasizes that microbiota affect host energy balance not only through caloric salvage but also via fermentation-derived metabolites particularly SCFAs [20]. These metabolites signal to host pathways regulating appetite, insulin sensitivity, and adipose tissue function [20]. SCFA, primarily acetate, propionate, and butyrate, are produced by microbial fermentation of indigestible carbohydrates. They serve as key ligands for free fatty acid receptors, thereby modulating local and systemic metabolic responses [21]. Obesity-associated microbial dysbiosis involves a reduced abundance of SCFA producing taxa, such as *Bifidobacterium* and *Faecalibacterium,* leading to decreased fecal and circulating SCFA levels [22]. This reduction impairs beneficial signaling and contributes to metabolic dysregulation, especially in childhood obesity, where these alterations correlate with increased body mass index (BMI) and disrupted energy utilization pathways [22]. Complementing these observations, inulin supplementation in children with obesity enriches SCFA-related metabolites and alters gut microbiota profiles associated with brain function, indicating potential effects on metabolic and neurobehavioral regulation [23]. Furthermore, recent study using mouse models demonstrate that fiber interventions increase SCFA-producing bacteria and enhance microbial functional capacity, contributing to improved metabolic outcomes [24]. SCFAs primarily exert antiobesogenic effects by stimulating enteroendocrine secretion of glucagon-like peptide-1 (GLP-1) and peptide YY (PYY), which suppress appetite through the gut-brain axis and enhance metabolic parameters, including insulin sensitivity [25]. Additional studies support the contribution of SCFAs, such as butyrate and propionate, in preventing body weight gain by modulation of gut microbiota, and enhances energy balance in HFD-induced obesity [26]. Dietary fiber enrichment in mice similarly reshapes gut microbiota toward SCFA-producing communities, increases microbial butyrate-production pathways, elevates fecal SCFA levels, and improves metabolic parameters under obesogenic conditions [27].

## METABOLIC ENDOTOXEMIA AND GUT-BARRIER INTEGRITY

Beyond the beneficial signaling roles of microbial metabolites, dysbiosis has been implicated in the impairment of intestinal barrier integrity. Disruption of epithelial tight junction proteins, including occludin and zonula occludens-1 (ZO-1), together with thinning of the mucus layer, increases intestinal permeability (“leaky gut”) and facilitates the translocation of bacterial components such as lipopolysaccharides (LPS) into the portal circulation [20,28]. This process, commonly referred to as metabolic endotoxemia, is characterized by a chronic low-grade inflammatory state that exacerbates obesity-related metabolic disturbances. Dysbiosis, often marked by expansion of Gram-negative taxa such as Proteobacteria and reduction of barrier-supporting microbes, enhances LPS exposure to Toll-like receptor 4 (TLR4), triggering nuclear factor (NF)-κB activation and the release of pro-inflammatory cytokines including tumor necrosis factor (TNF)-α and interleukin (IL)-6 [29,30]. HFD amplifies this pathway by promoting LPS-producing bacteria, reducing tight junction protein expression, and increasing circulating endotoxin levels, ultimately contributing to adipose tissue inflammation, hepatic steatosis, and insulin resistance through the gut–liver axis [31,32]. Both preclinical and human studies support this mechanism: short-term high-fat feeding in older adults with obesity increases circulating endotoxins and zonulin, linking dietary fat intake to barrier dysfunction and systemic inflammation [33^▪^]. Conversely, interventions that preserve epithelial integrity, such as citral supplementation or essential amino acids that support mitochondrial function, reduce LPS translocation and improve metabolic outcomes [30,32]. Emerging evidence also suggests sex-specific differences, with greater barrier vulnerability and inflammatory amplification reported in females [28]. Collectively, these findings position metabolic endotoxemia as a central mediator of dysbiosis-driven metabolic dysfunction in obesity.

## DYSREGULATED ENDOCRINE SIGNALLING IN DIET-INDUCED OBESITY

Enteroendocrine signaling is regulated through both microbiota-independent and microbiota-dependent mechanisms. Enteroendocrine hormones such as glucagon-like peptide-1 (GLP-1) and PYY can be acutely stimulated by luminal nutrients via established nutrient-sensing pathways [34], and these responses are preserved in germ-free conditions. However, germ-free and microbiota-depleted models demonstrate that the magnitude, kinetics, and downstream metabolic effects of these responses are altered in the absence of microbial metabolism, highlighting a modulatory role for microbiota-derived metabolites such as SCFAs and bile acid derivatives [35,36]. These pathways therefore operate in a complementary and context-dependent manner. In addition to inflammatory and metabolic effects, gut microbiota dysbiosis contributes to obesity through disruption of endocrine signaling along the gut–brain axis [5,37^▪^]. Diet-induced microbial alterations impair enteroendocrine hormone secretion, appetite regulation, and energy homeostasis. Changes in bile acid composition represent an early mechanism in obesity-prone states. Reduced levels of glycodeoxycholic acid (GDCA) limit activation of the Takeda G protein-coupled receptor 5 (TGR5), thereby suppressing ileal GLP-1 secretion and brown adipose tissue thermogenesis. These alterations precede overt obesity and contribute to early glycolipid dysregulation [37^▪^], highlighting microbial bile acid signaling deficits as a key driver of endocrine imbalance.

Experimental interventions support this mechanistic link. In HFD-induced obese mice, probiotic bacterial strains such as *Lacticaseibacillus rhamnosus* HF01 increases short-chain fatty acid production, enhances PYY secretion, downregulates hypothalamic neuropeptide Y (NPY), upregulates pro-opiomelanocortin (POMC), and suppresses appetite via gut–brain axis modulation [5]. Similarly, *Lactobacillus rhamnosus* GG-derived supernatant restores GLP-1 secretion, reduces lipotoxic stress in intestinal L cells, and improves glucose tolerance by reshaping microbial and enteroendocrine function [38]. Human and preclinical studies further demonstrate bidirectional gut–brain–endocrine interactions. Although hypothalamic leptin signaling can acutely modulate gut microbiota composition, this regulatory feedback is attenuated in diet-induced obesity, disrupting sympathetic activation and hormonal balance [39]. Pharmacological interventions, such as orlistat administration, also alters microbial composition and improves GLP-1-related pathways, suggesting partial reversibility of endocrine dysfunction [40]. Collectively, these findings indicate that diet-induced dysbiosis promotes obesity not only through inflammation but also through impaired enteroendocrine signaling and disrupted bile acid–hormone crosstalk. While enteroendocrine hormone secretion can occur independently of the microbiota through direct nutrient sensing, diet-induced dysbiosis alters the magnitude and downstream metabolic effects of these responses via microbiota-derived metabolites, particularly under chronic obesogenic conditions. Targeting microbial and/or endocrine pathways therefore represents a promising strategy to restore hormonal homeostasis and counteract obesity progression.

## INTEGRATED MECHANISMS: FROM MICROBIAL DYSBIOSIS TO ADIPOSE TISSUE AND HEPATIC DYSFUNCTION

The functional consequences of diet-induced dysbiosis extend beyond altered energy extraction and endocrine signaling to sustained changes in adipose tissue and hepatic metabolism, where microbial metabolites, inflammatory mediators, and hormonal disturbances converge to reinforce the obese phenotype. In adipose tissue, microbiota-derived signals regulate inflammation, adipogenesis, and thermogenic capacity. In HFD-fed mice, curcumin supplementation reshapes the gut microbiota toward anti-inflammatory profiles, reduces visceral adiposity, attenuates macrophage infiltration and pro-inflammatory cytokine expression, and promotes white adipose tissue browning with increased uncoupling protein 1 (UCP1) expression and thermogenesis [41]. Similarly, interventions that enhance SCFAs availability or restore bile acid signaling stimulate brown adipose tissue activity, improve insulin sensitivity, and mitigate adipose inflammation [37^▪^,^42^].

The liver represents another primary target of microbiota-driven pathology through the gut–liver axis. Dysbiosis promotes hepatic steatosis by facilitating lipopolysaccharide translocation and disrupting bile acid–mediated regulation of lipid metabolism. In obesity-resistant mice exposed to an HFD, distinct microbial configurations limit intestinal fatty acid absorption and prevent hepatic triglyceride accumulation, underscoring the microbiota's role in hepatic fat partitioning [12]. Microbiota adapted to dietary fiber similarly reduce fructose-driven steatosis, insulin resistance, and fibrosis by suppressing de novo lipogenesis and strengthening gut barrier function [43^▪▪^]. Conversely, transplantation of obesity-associated microbiota accelerates hepatic inflammation and fibrotic progression via activation of the TLR4–NF-κB–mTOR pathway, providing direct evidence of causality within the gut–liver axis [44]. Consistently, multiomics analyses across intestinal, hepatic, and adipose tissues identify microbial signatures linked to systemic glucose dysregulation, inflammation, and lipid metabolism [45^▪▪^].

Collectively, these findings highlight the integrated and tissue-specific metabolic consequences of diet-induced dysbiosis in obesity.

## TARGETING THE GUT MICROBIOTA IN OBESITY

The gut microbiota is increasingly recognized as a modifiable therapeutic target in obesity, influencing metabolic signaling and inflammation through microbial metabolites and bile acid pathways. Microbiota-targeted strategies, ranging from direct fecal microbiota transplantation (FMT) to indirect approaches such as prebiotics, synbiotics, and dietary modulation, are emerging as potential adjuvants to conventional treatments, including diet, exercise, and GLP-1 receptor agonists. FMT from lean or metabolically healthy donors provides a direct method to reshape microbial composition and function. In preclinical models, transplantation of microbiota from semaglutide-treated obese mice transferred antiobesity effects to recipients, improving insulin sensitivity and inducing shifts in amino acid and pyrimidine metabolism [46]. Human data further support this approach. A four-year follow-up of a double-blind randomized controlled trial in adolescents with obesity demonstrated that a single FMT resulted in durable donor-derived bacterial and bacteriophage engraftment, accompanied by sustained improvements in waist circumference, body fat percentage, metabolic syndrome severity, systemic inflammation, and HDL cholesterol, despite no significant change in adjusted body mass index [18^▪▪^].

Indirect microbiota modulation offers accessible, noninvasive alternatives. Multimodal dietary interventions incorporating prebiotics, time-restricted eating, and high-fiber components increase microbial alpha diversity and enrich beneficial taxa such as *Faecalibacterium*. These changes are associated with greater reductions in fat mass and visceral adiposity compared with standard dietary approaches [47]. In pediatric obesity, inulin supplementation altered gut–brain axis metabolites, including putrescine, spermine, and tyrosine, linking microbial shifts to appetite and energy regulation [23]. A 12-week randomized controlled trial of synbiotic supplementation in overweight and obese adults demonstrated a modest but significant reduction in visceral adipose tissue, particularly among men and individuals with moderate overweight, alongside transient increases in *Bifidobacterium animalis* subsp. *lactis* and *Lactobacillus rhamnosus*[48]. Similarly, resistant starch supplementation promoted weight loss and transferable metabolic benefits in mouse FMT models by modulating bile acids and restoring intestinal barrier integrity [49^▪^]. Evidence from human intervention studies supporting these approaches is summarized in Table 1.

**Table 1:** Human intervention studies targeting the gut microbiota to improve obesity-related metabolic outcomes

Intervention type	Population	Duration	Main outcomes	Key microbiota/metabolic changes	Reference
FMT	Adolescents with obesity	4 years	↓ adiposity, ↓ inflammation, ↑ HDL; no BMI change	Durable donor-derived bacterial and bacteriophage engraftment	[18^▪▪^]
Prebiotic (inulin)	Children with obesity	6 months	Modulation of appetite-related metabolites	Changes in metabolites (putrescine, spermine, tyrosine)	[23]
Prebiotic (chicory root fiber)	Adults with obesity	12 weeks	↑ insulin sensitivity	Enhanced colonic butyrate production	[27]
Prebiotic (resistant starch)	Adults with overweight/obesity	8 weeks	Weight loss, improved metabolic profile	Reshaping of gut microbiota; improved bile acid metabolism	[49^▪^]
Synbiotic	Overweight/obese adults	12 weeks	↓ visceral adiposity	Transient increases in *Bifidobacterium animalis* subsp. *lactis* and *L. rhamnosus*	[48]
Multimodal dietary intervention	Adults with obesity	6 months (active weight loss phase)	↓ fat mass, ↓ visceral adiposity	Increased microbial alpha diversity and beneficial taxa	[47]

## CONCLUSION

Recent advances have shifted from taxonomic descriptions of dysbiosis toward a functional framework in which diet-induced microbial alterations disrupt metabolite signaling, barrier integrity, and enteroendocrine regulation, thereby amplifying metabolic inflammation and impairing metabolic flexibility. Rather than acting as an isolated driver of obesity, the gut microbiota appears to modulate host susceptibility to obesogenic environments through integrated gut–brain and gut–liver pathways. Although microbiota-targeted interventions demonstrate potential to restore specific metabolic functions, their clinical translation remains limited by interindividual variability, incomplete mechanistic understanding, and limited long-term data. Moving forward, integrating functional microbiome profiling with host metabolic phenotyping may facilitate precision strategies that target microbial pathways to complement established therapeutic approaches in obesity.

## Acknowledgements

*We thank all members of the Al Nabhani laboratory for their support.*

### Financial support and sponsorship

*Z.A.N. is supported by the European Research Council Starting Grant (WePredict: 949613), Swiss National Science Foundation (SNSF, grants number: 238856; 213452; 215675; 222781), Inselspital, Helmut Horten Stiftung (Project ID: 2021-YIG-083), Swiss Cancer Research Foundation (KFS-5691-08-2022), Kenneth Rainin Foundation, Crohn's Colitis Foundation of America, Dementia Research Switzerland – Synapsis Foundation, along with the Heidi Seiler-Stiftung, Alzheimer's Association (AARG-22–974406), Ruth & Arthur Scherbarth Stiftung, Novartis Foundation for Medical-Biological Research, Edoardo R.-, Giovanni, Giuseppe und Chiarina Sassella-Stiftung, Jubiläumsstiftung von Swiss Life, and a research award from the Biostime Institute for Nutrition & Care.*

### Conflicts of interest

*The authors declare no conflict of interest.*

*Declaration of generative AI and AI-assisted technologies in the writing process: During the preparation of this work the authors used [ChatGPT 5] in order to polish the writing to improve the spelling, grammar, clarity, concision and overall readability. After using this tool, the authors reviewed and edited the content as needed and take full responsibility for the content of the publication.*

## REFERENCES AND RECOMMENDED READING

Papers of particular interest, published within the annual period of review, have been highlighted as:

▪ of special interest
▪▪ of outstanding interest
